# 

**Published:** 1898-04

**Authors:** 


### EDITORIAL.

### Tri-State Meeting.

The State Dental Societies of Ohio, Michigan and Indiana will hold a joint meeting, according to former agreement, on June 21st, 22d and 23rd, instead of a week earlier, as was through misinformation, stated in the March number of this journal, at Put-in-Bay, Ohio.

The programme is about completed, and will be issued in a few days. It is greatly to be desired that every dentist who has an interest in the welfare and progress of the profession within these three States will be present, and, indeed, from the surrounding States as well.

The indications are that the meeting will be a large and interesting one. Arrangements are being made for an extensive clinic, in which many new and interesting things will be presented, and those who were at the Detroit meeting, three years ago, will certainly have a desire to enjoy another such convocation. Let arrangements be made by every one interested, with a view of being present at that time and place.

### Matrimonial.

Married at Fargo, S. Dakota, on the evening of January 6th, 1898, Dr. C. R. Butler, of Cleveland, Ohio, to Mrs. J. B. Eddy, of Fargo.

This procedure will meet with the hearty approval of Dr. Butler's friends, of whom he has a host. They will rejoice with him and his good wife in the happy union they have formed. May this blessed companionship be one that will grow brighter and more delightful as the journey along life's pathway is traveled by these dear friends.

### The Eastern Indiana Dental Association.

The tenth annual meeting of this society will be held in Muncie, Indiana, May 3d and 4th, 1898. This is one of the active and efficient societies of Indiana. These meetings are always interesting and instructive, with a good admixture of the social element. Ample preparation has been made for a series of clinics. A cordial invitation is extended to all honorable men of the profession outside of the territory and the society, and it is earnestly hoped that many such will be present, as they always add to the interest of the occasion. Any desiring information regarding this meeting, address Dr. H. M. Thompson, Greensburg, Indiania.

### Bibliographical.

The second volume of the great work of Dr. John N. Farrar, M.D., D.D.S., has just been issued from the press, and will answer an earnest expectation which has been entertained in regard to it for the last two or three years.

We have not had opportunity to examine the work, but judging from the first volume and from Dr. Farrar's great ability and indefatigable energy bestowed on the subject of "Irregularities of the Teeth," the second volume will be equal, if not superior, to the first in value to the dentist. Dr. Farrar has devoted more time, energy and work upon this subject than any other living man, and his work, complete, will be the leading work of the world on this subject.

After a careful examination of the work, we will be prepared to speak more in detail in regard to it.

### The National Dental Association—Division of the East.

It is proposed to hold a meeting at Albany, New York, on Thursday, the 12th of May, 1898, at 2 o'clock, to organize a branch of the National Dental Association, and to transact any other business which may properly come to them. This division embraces New York, New Jersey, Pennsylvania; Massachusetts, Ohio, Indiana, and the other Eastern States.

All members of the National Dental Association are eligible to membership in this body, and it is hoped that all members of the profession, so far as practicable, will make it a point to be present at that time, and aid in the organization. Railroad tickets, at reduced rates, for this occasion are obtainable for all going to this meeting.

### CONTENTS.

COMMUNICATIONS.

### THE DENTAL REGISTER,

**A Monthly Magazine Devoted to the Interests of the**

### DENTAL PROFESSION.

*Terms Reduced to* $2.00 *Per Year. Single Copies,* 25 *Cents.*

**EDITED BY PROF. J. TAFT, D.D.S.**

—AND—

**Published by SAM'L A. CROCKER & CO., Ohio Dental and Surgical Depot.**

The volume commences in January and closes in December.

The Register being issued monthly is always fresh, therefore Indispensable to the practitioner who desires to keep posted up with the new inventions and improvements which are daily developed. Neither expense nor effort will be spared to give value and variety to its contents. Articles will be illustrated with well executed engravings whenever they will add to the interest or assist to explanation.

Its extensive circulation and frequency of issue make it one of the BEST DENTAL ADVERTISING MEDIUMS in the United States.

All subscriptions must begin with January or July.

Local or special notices, five lines or less, will be inserted for $3 each insertion; over five lines, 50 cts. per line.

All advertising bills payable in advance, or at furthest, after the issue of the first number containing the advertisement. All transient advertisements to be paid for in advance.

☞CONTRIBUTIONS SOLICITED.

Exclusively Editorial Communications should be addressed to the Editor.

The latest number of the Register may be ordered with or without other good[illegible] any time, and all letters should be addre[illegible]

